# Efficacy and Safety of Iguratimod for the Treatment of Rheumatoid Arthritis

**DOI:** 10.1155/2013/310628

**Published:** 2013-11-26

**Authors:** Jiangtao Li, Hejuan Mao, Yan Liang, Yanrong Lu, Shuo Chen, Nanping Yang, Guixiu Shi

**Affiliations:** ^1^West China Hospital, Sichuan University, Chengdu, Sichuan 610041, China; ^2^Department of Rheumatology and Immunology, The First People's Hospital of Yibin, Yibin, Sichuan 644000, China; ^3^Department of Otolaryngology, The First People's Hospital of Yibin, Yibin, Sichuan 644000, China; ^4^The Chinese Cochrane Center, West China Hospital, Sichuan University, Chengdu, Sichuan 610041, China; ^5^Department of Rheumatology and Clinical Immunology, The First Affiliated Hospital of Xiamen University, Xiamen 361003, China

## Abstract

All randomized controlled trials (RCTs) of iguratimod for rheumatoid arthritis (RA) to assess its efficacy and safety are included in this paper. The Review Manager software was used for meta-analysis to assess risk bias of the studies included, and GRADE profiler software was used for the evidence quality of the studies included. Four RCTs involving 1407 patients with RA were included. Meta-analyses showed that, after 24-week therapy, ACR20, tender joint count, swollen joint count, rest pain, physician and patient global assessment of disease activity, HAQ score, ESR, and CRP in iguratimod group were better than those in placebo group and that the difference between those of iguratimod group and those of other DMARDs (MTX and SASP) group was not significant. GRADE evidence classification of the studies included was moderate. Iguratimod for RA had few adverse events, and its efficacy and safety were the same as those of MTX and SASP for RA. The results of this systematic review suggest that more high-quality and large-scaled RCTs were needed to determine the efficacy of iguratimod for RA and whether iguratimod is as effective as other DMARDs besides MTX and SASP.

## 1. Introduction

Rheumatoid arthritis (RA) is chronic systemic inflammatory and autoimmune disease of unknown etiology that primarily targets synovial tissue and is characterized by an activation of T lymphocyte, an increase in interleukin and tumor necrosis factor, and severe chronic inflammation of the joints, resulting in erosion and destruction of cartilage, bone, and tendon [1, 2]. It is relatively common, with a prevalence of slightly less than 1% in adults all over the world [1]. Prevalence of moderate and severe disability in adults aging over 60 (in millions) due to rheumatoid arthritis by leading health condition associated with disability is 1.7 in high-income countries and 3.7 in low- and middle-income countries in 2012 [3]. Years lost due to disability (YLD) per 100 000 adults aging over 60 due to rheumatoid arthritis is the 11th in the world in 2012 [3]. Current treatments for RA emphasize the early use of traditional disease-modifying antirheumatic drugs (DMARDs), such as methotrexate (MTX), salazosulfapyridine (SASP), leflunomide, and cyclophosphamide to minimize or prevent joint damage. In addition, biologic agents such as necrosis factor-*α* blocker, anti-interleukin antibody, and CD20 monoclonal antibody are also used to teat RA. In the recent ten years, iguratimod (T-614) has been used to treat RA as a novel immunomodulator. It functions by suppressing the production of some inflammatory cytokines, including interleukin-1 (IL-1), IL-4, IL-6, IL-17, tumor necrosis factor, nuclear factor-kappaB, and interferon *in vitro* (synovial cells and some cell lines) and *in vivo* (mouse models) [4, 5]. Iguratimod also reduced immunoglobulin production by acting directly on human B lymphocytes without affecting B lymphocyte proliferation [6]. Direct evidence has also showed that iguratimod can dramatically suppress disease progression and markedly protect affected joints against cartilage destruction and bone erosion in collagen-induced arthritis rats [5]. In addition, iguratimod decreased production of matrix metalloproteinases (MMP-1 and MMP-3) and inhibits the migratory expansion of rheumatoid synovial fibroblasts *in vitro* [7]. Although iguratimod has been used to treat RA for 10 years since 2003, no systematic review has been done on its efficacy and safety. Therefore, we conducted this systematic review to assess the efficacy and safety of iguratimod (T-614) for RA.

## 2. Materials and Methods

### 2.1. Types of Studies and Inclusion and Exclusion Criteria

#### 2.1.1. Types of Studies

All randomized clinical trials (RCTs) were published in all journals, with a minimum duration of study of at least six months (or 24 weeks).

#### 2.1.2. Types of Participants

*(1) Inclusion Criteria.* Patients with clinical diagnosis of rheumatoid arthritis (RA) of all eligible RCTs according to the American Rheumatism Association (ARA) criteria are enrolled within the inclusion criteria. Age of patients with only RCTs was at least 18 years old, and sex, race, and region of patients were not limited. These patients must have active disease as shown in the following outcomes: (a) ACR20; (b) tender joint count (TJC); (c) swollen joint count (SJC); (d) assessment of rest pain; (e) physician global assessment of disease activity; (f) patient global assessment of disease activity; (g) health assessment questionnaire (HAQ) score; (h) erythrocyte sedimentation rate (ESR); (i) C-reactive protein (CRP); and (j) adverse events reports. 

*(2) Exclusion Criteria.* The studies on patients with both RA and cancer, abnormal hepatic dysfunction or renal dysfunction, or pregnant women and patients with diabetes mellitus, hypertension, or abnormal function of gastrointestinal tract were excluded. The studies of duplicate records, non-RCTs, nonclinical trials, the same study, ongoing without outcomes reported, or no full text were excluded.

#### 2.1.3. Types of Intervention

Studies comparing iguratimod treatment (as monotherapy or in combination with other DMARDs) at a dose of 25 or 50 mg/day with placebo or other DMARDs were included. The duration of treatment in the trials must have been at least six months (or 24 weeks).

#### 2.1.4. Types of Outcome Measures

*(1) Primary Outcomes.* Primary outcome measures were those defined as the ACR core set of disease activity measures for RA for clinical trials, which were endorsed by EULAR and the Outcome Measures in Rheumatology Clinical Trials (OMERACT) [8, 9]. They included (1) tender joint count; (2) swollen joint count; (3) assessment of rest pain; (4) patient global assessment of disease activity; (5) physician global assessment of disease activity; (6) health assessment questionnaire (HAQ) score; (7) acute phase reactants (ESR and CRP); and (8) radiographic change of bone and joint damage for trials lasting at least one year. In addition, the numbers of patients who met the ACR20, ACR50, and ACR70 response criteria were included. The EULAR response criteria are measured as the Disease Activity Score (DAS) according to the EULAR response criteria [10]. 

*(2) Secondary Outcomes.* Secondary outcome measures included health-related quality of life (HRQoL) of the patients, reported side effects, total number of patients withdrawn from the studies, and withdrawals due to adverse events.

We defined serious adverse events according to the ICH Guidelines [11] as any event that led to death, that was life-threatening, and required in-patient hospitalization or prolongation of existing hospitalization, and that resulted in persistent or significant disability and as any important medical events, which might have jeopardized the patient or required intervention to prevent them. We did not consider other adverse events to be serious.

### 2.2. Search Methods for Identification of Studies

#### 2.2.1. Electronic Search

We searched the Cochrane Central Register of Controlled Trials (the Cochrane Library, 11 July 2012), PubMed (1950 to 29 March 2013), Embase (1980 to 29 March 2013), the Chinese Biomedical Database (CBM) (1975 to 18 March 2013), China National Knowledge Infrastructure (CNKI) (1917 to 18 March 2013), VIP Database (1989 to 18 March 2013), and the WHO ICTRP (18 March 2013).

#### 2.2.2. Search Strategy

Search strategy for the Cochrane Library, PubMed, Embase, and so forth, in English is given as follows:

 #1 iguratimod
 #2 T-614
 #3 CAS 123663-49-0
 #4 C17H14N2O6S
 #5 Ailamode
 #6 N-3-formylamino-4-oxo-6-phenoxy-4H-chromen-7-yl-methanesulfonamide
 #7 3-formylamino-7-methylsulfonylamino-6-phenoxy-4H-1-benzopyran-4-one
 #8 or/#1–7
 #9 randomized controlled trials
 #10 random
 #11 control
 #12 trials
 #13 or/#9–12
 #14 rheumatoid arthritis
 #15 (#8, #13, and #14).

We searched the Chinese Biomedical Database (CBM), China National Knowledge Infrastructure (CNKI), and VIP Database by using the strategy adjusted in Chinese.

### 2.3. Data Collection and Analysis

#### 2.3.1. Selection of Studies

Three review authors (Jiangtao Li, Hejuan Mao, and Shuo Chen) independently assessed for inclusion all of the potential studies identified as a result of the search strategy. We resolved disagreements through discussion. We included only those studies that used a strict randomization procedure. We telephoned or wrote letter by email to contact the authors of those articles in which “randomly allocated participants” was mentioned to determine whether the randomization procedure was adequate or not.

#### 2.3.2. Data Extraction and Management

We designed a form to extract data for retrieval of records to meet the needs of the project design. For eligible studies, the three review authors extracted the data using the agreed form. We resolved discrepancies through discussion. Where disagreement could not be resolved even through discussion, experts in the area were contacted to make a decision. We input the data into Review Manager software (RevMan Manager version 5.2.4, 2013) and checked them for accuracy.

#### 2.3.3. Assessments Risk of Bias in Included Studies and Quality of Evidence

According to assessing risk of bias in included studies in the Cochrane Handbook for Systematic Reviews of Interventions (version 5.1.0, updated in March 2011) [12], we assessed each study included in random sequence generation and allocation concealment (selection bias), blinding (performance bias and detection bias), incomplete outcome data (attrition bias), selective reporting (reporting bias), and other biases. According to GRADE Handbook for grading quality of evidence and strength of recommendation, version 3.2 (updated in March 2009) [13] and GRADE profiler 3.6 software, we assessed quality of evidence of each study included.

#### 2.3.4. Methods of Statistical Analysis

Statistical analysis was made by using the Review Manager software (Cochrane Collaboration's RevMan 5.2.4, 2013). For continuous variables, mean differences (MD) or standardized mean differences (SMD) were used to describe effect size with confidence interval (CI) set at 95%. For dichotomous variables, odds ratio (OR), relative risk (RR), and risk difference (RD) were used to describe effect with confidence interval (CI) set at 95%.  *χ* ^2^  test was used to analyze the heterogeneity among results. Where there is no heterogeneity (*P* > 0.1; *I* ^2^< 50%), the fixed effects model analysis was made. If there is heterogeneity between studies, random effects model was used, and the source, cause, and sensitivity of heterogeneity were analyzed in subgroups. Where there is clinical heterogeneity between studies, descriptive analysis was made.

## 3. Results and Discussion

### 3.1. Results

#### 3.1.1. Results of the Search

**Figure 1:**
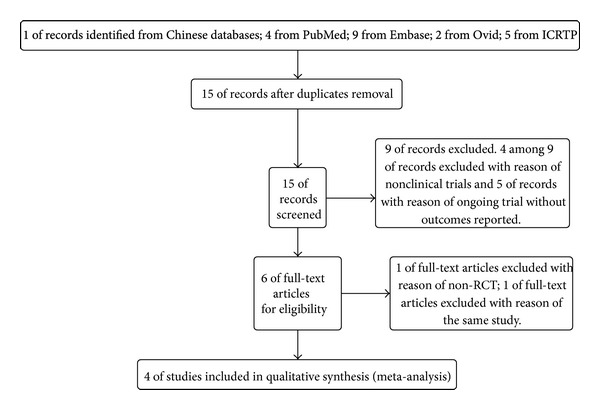
Search study flow diagram.

We identified 21 records by electronic searches and hand searches, including 1 of records indentified from the Chinese database, four from PubMed, nine from Embase, two from Ovid, and five from ICRTP. We excluded 17 records because of duplicate records, non-RCTs, nonclinical trials, the same study, ongoing without outcomes reported, or no full text. At the end, we finalized four studies [2, 14–16] to make quantitative synthesis (meta-analysis) (Figure 1).

#### 3.1.2. Characteristics of Included Studies (Table 1)

*(1) Types of Studies.* All of the four included studies were multicenter, double-blind, randomized controlled trials. Three of them had a placebo control group. The duration of these studies was about six months (from 24 weeks to 28 weeks). The studies were conducted in China (*n* = 2) and Japan (*n* = 2). 

*(2) Participants of Included Studies.* In the four RCT studies included [2, 14–16], in total, 1407 patients with RA were enrolled (1159 females and 248 males; mean age was from 45.9 to 58.2 years old). Diagnosis of RA was based on the American Rheumatism Association (ARA) criteria in 1987 [17] in the two Japanese studies [15, 16] and on the American Rheumatism Association (ARA) criteria in 1991 [18] in the two Chinese studies [2, 15]. 

*(3) Interventions of Studies Included.* Among the four studies included [2, 14–16], four different types of intervention were used: (a) iguratimod versus placebo and iguratimod versus SASP [14], (b) iguratimod versus placebo [2], (c) iguratimod versus MTX, and (d) iguratimod + MTX versus placebo + MTX [16]. The low dose of MTX (6–8 mg per week) was in the study of iguratimod + MTX versus placebo + MTX, and the low dose of MTX (15 mg per week) was in the study of iguratimod versus MTX. Duration of treatment was 24 weeks [2, 15, 16] and 28 weeks in one study [14]. 

*(4) Measures of Outcomes.* Primary outcomes (ACR20) and secondary outcomes (tender joint count; swollen joint count; assessment of rest pain; physician global assessment of disease activity; patient global assessment of disease activity; HAQ score; ESR; CRP; adverse events, etc.) were all reported in the four studies included.

#### 3.1.3. Assessment of Methodological Quality of  Studies Included [13] (Figures 2 and 3)

*(1) Randomized Method and Allocation Concealment.* In all of the four studies included [2, 14–16], randomization was described. In two studies [2, 15], random sequence generation and allocation concealment were detailed, and the validity of method was ascertained by telephone. In the other two studies [14, 16], random sequence generation and allocation concealment were described but not ascertained because the author could not be reached via email. For this reason, some degree risk of selection bias of two studies included [15, 16] existed. 

*(2) Blinding of Participants and Personnel.* In all of the four studies [2, 14–16] blinding of participants and personnel was described. Double blinding was detailed in three studies [2, 14, 15] and not mentioned in one study [16]. The author could not be reached via email. Thus, low risk of performance bias and detection bias of outcome assessment existed. 

*(3) Incomplete Outcome Data.* Withdrawals, dropouts, loss of followup, and intent-to-treat analysis were reported in detail in two studies included [2, 15] and were not described in the other two studies included [14, 16]. Therefore, high risk of attrition bias in general existed. 

*(4) Selective Reporting Bias.* The four studies [2, 14–16] included reported all outcomes, including adverse events. There was no selective reporting bias. 

*(5) Other Biases.* Baseline information including sex, age and secondary outcomes of the four studies [2, 14–16] was described in detail, and outcomes of different groups had robust comparability. Sample sizes of three studies [2, 15, 16] were estimated. All of the studies included were conducted in general hospitals or good clinical practice bases of medical universities, with all of which having high scientific research level and thus reducing other risk biases of these studies to a large extent.

#### 3.1.4. Meta-Analysis

*(1) Primary Outcomes*

**Figure 4:**
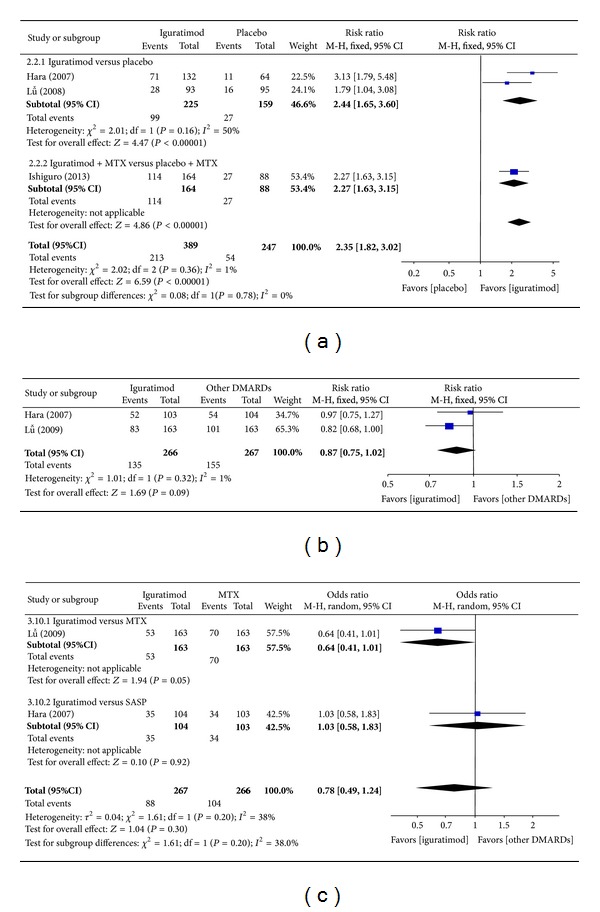
(a) Comparison of ACR20 at 24 weeks between iguratimod and placebo groups. (b) Comparison of ACR20 at 24 weeks between iguratimod and other DMARDs (MTX and SASP) groups. (c) Comparison of ACR50 at 24 weeks between iguratimod and other DMARDs (MTX and SASP) groups.

*(a) ACR20, ACR50, and ACR70 at 24 Weeks.* Superiority pooled analysis of three studies [2, 14, 16] included shows that ACR20 at 24 weeks among patients with RA in the iguratimod group was significantly superior to that of the placebo group (RR = 2.35, 95% CI: 1.82, 3.02 in Figure 4(a)). Noninferiority pooled analysis of two studies [14, 15] included showed that ACR20 at 24 weeks among patients with RA in iguratimod group did not differ significantly from that of other DMARDs (MTX and SASP) group (RR = 0.87, 95% CI: 0.75, 1.02 in Figure 4(b)). Only in one study [2], ACR50 at 24 weeks did not differ significantly between the iguratimod group and the placebo group (RR = 0.62, 95% CI: 0.21, 1.82), and ACR70 at 24 weeks differed significantly (RR = 0.06, 95% CI: 0.01, 0.51). ACR50 at 24 weeks did not differ significantly between the iguratimod group and the other DMARDs (MTX and SASP) group [14, 15] (RR = 0.78, 95% CI: 0.49, 1.24 in Figure 4(c)). Only in one study [15], ACR70 at 24 weeks did not differ significantly between the iguratimod group and the other DMARDs (MTX) group (RR = 0.75, 95% CI: 0.43, 1.32).

**Figure 5:**
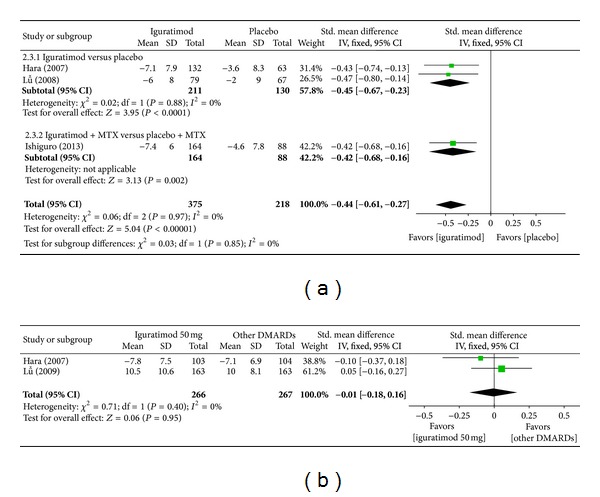
(a) Comparison of tender joint count between iguratimod and placebo groups. (b) Comparison of tender joint count between iguratimod and other DMARDs (MTX and SASP) groups.

*(b) Tender Joint Count.* Superiority analysis of three studies [14] included showed that tender joint count among patients with RA in the iguratimod group reduced more than that of the placebo group with a significant difference (MD = −0.44, 95% CI: −0.61, −0.27 in Figure 5(a)). Noninferiority analysis of two studies [14, 15] included showed that tender joint count among patients with RA in the iguratimod group and in the other DMARDs (MTX and SASP) group did not differ significantly (MD = −0.01, 95% CI: −0.18, 0.16 in Figure 5(b)). 

**Figure 6:**
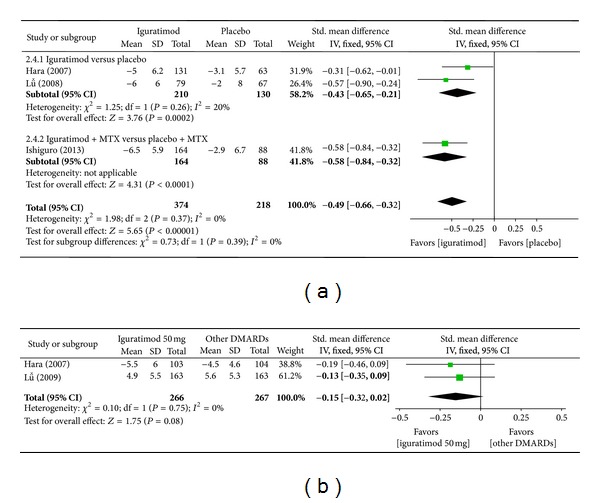
(a) Comparison of swollen joint count between iguratimod and placebo groups. (b) Comparison of swollen joint count between iguratimod and other DMARDs (MTX and SASP) groups.

*(c) Swollen Joint Count.* Superiority analysis of three studies [2, 15, 16] included showed that swollen joint count among patients with RA in the iguratimod group reduced more than that of the placebo group with a significant difference (MD = −0.49, 95% CI: −0.66, −0.32 in Figure 6(a)). Noninferiority analysis of two studies [14, 15] included showed that swollen joint count among patients with RA in the iguratimod group and in the other DMARDs (MTX and SASP) group did not differ significantly (MD = −0.15, 95% CI: −0.32, 0.02 in Figure 6(b)). 

**Figure 7:**
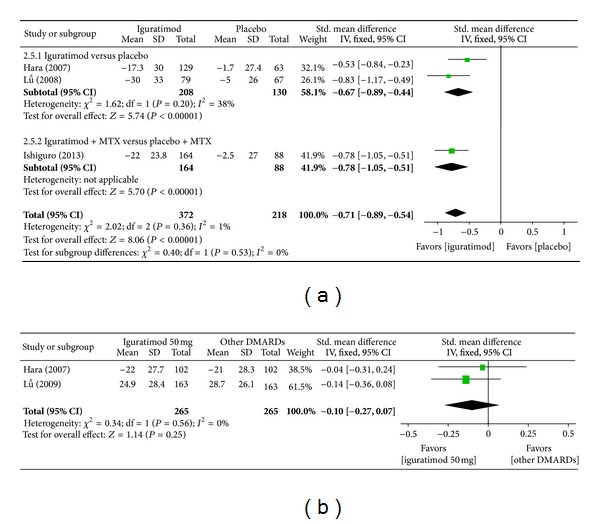
(a) Comparison of assessment of rest pain between iguratimod and placebo groups. (b) Comparison of assessment of rest pain between iguratimod and other DMARDs (MTX and SASP) groups.

*(d) Assessment of Rest Pain.* Superiority analysis of three studies included [2, 14, 16] showed that assessment of rest pain among patients with RA in the iguratimod group was worse than that of the placebo group with a significant difference (MD = −0.71, 95% CI: −0.89, −0.54 in Figure 7(a)). Noninferiority analysis of two studies included [14, 15] shows that assessment of rest pain among patients with RA in the iguratimod group and in the other DMARDs (MTX and SASP) group did not differ significantly (MD = −0.10, 95% CI: −0.27, 0.07 in Figure 7(b)). 

**Figure 8:**
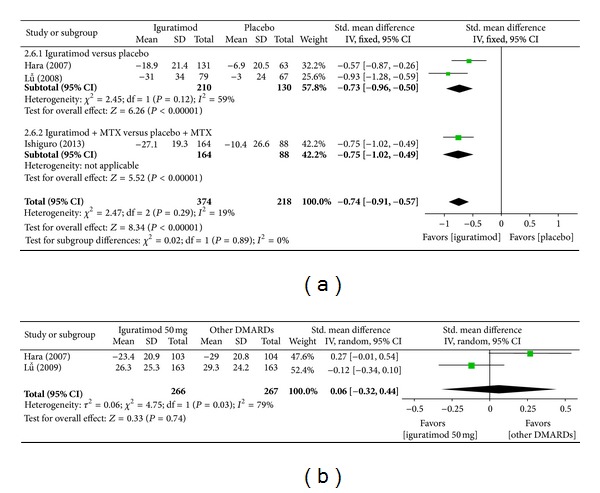
(a) Comparison of physician global assessment of disease activity between iguratimod and placebo groups. (b) Comparison of physician global assessment of disease activity between iguratimod and other DMARDs (MTX and SASP) groups.

*(e) Physician Global Assessment of Disease Activity.* Superiority analysis of three studies included [2, 15, 16] showed that physician global assessment of disease activity among patients with RA in the iguratimod group reduced more than that of the placebo group with a significant difference (MD = −0.74, 95% CI: −0.93, −0.57 in Figure 8(a)). Noninferiority analysis of two studies included [14, 15] showed that physician global assessment of disease activity among patients with RA in the iguratimod group and in the other DMARDs (MTX and SASP) did not differ significantly (MD = 0.06, 95% CI: −0.32, 0.44 in Figure 8(b)). 

**Figure 9:**
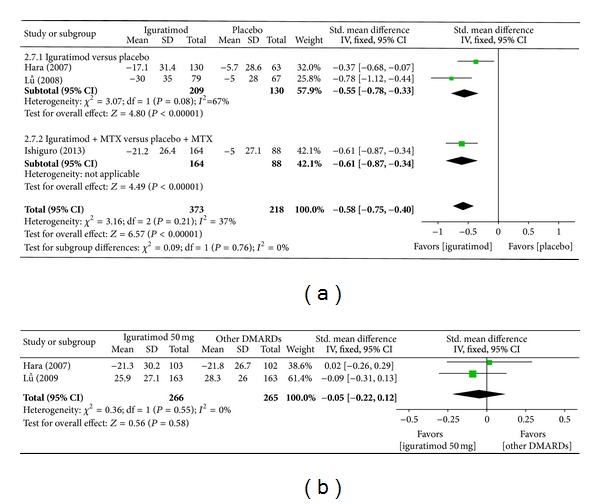
(a) Comparison of patient global assessment of disease activity between iguratimod and placebo groups. (b) Comparison of patient global assessment of disease activity between iguratimod and other DMARDs (MTX and SASP) groups.

*(f) Patient Global Assessment of Disease Activity.* Superiority analysis of three studies included [14–16] showed that patient global assessment of disease activity among patients with RA in the iguratimod group reduced more than that of placebo group with a significant difference (MD = −0.58, 95% CI: −0.80, −0.36 in Figure 9(a)). Noninferiority analysis of two studies included [14, 15] showed that patient global assessment of disease activity among patients with RA in the iguratimod group and in the other DMARDs (MTX and SASP) group did not differ significantly (MD = −0.05, 95% CI: −0.22, 0.12 in Figure 9(b)). 

**Figure 10:**
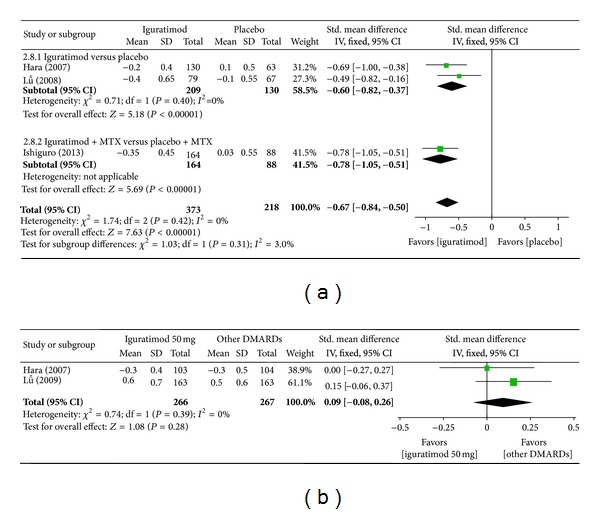
(a) Comparison of HAQ score between iguratimod and placebo groups. (b) Comparison of HAQ score between iguratimod and other DMARDs (MTX and SASP) groups.

*(g) HAQ Score*. Superiority analysis of three studies included [14–16] shows that HAQ score among patients with RA in the iguratimod group improved more than that of the placebo group with a significant difference (MD = −0.67, 95% CI: −0.84, −0.50 in Figure 10(a)). Noninferiority analysis of two studies included [14, 15] showed that HAQ score among patients with RA in the iguratimod group and in the other DMARDs (MTX and SASP) group did not differ significantly (MD = 0.09, 95% CI: −0.08, 0.26 in Figure 10(b)). 

**Figure 11:**
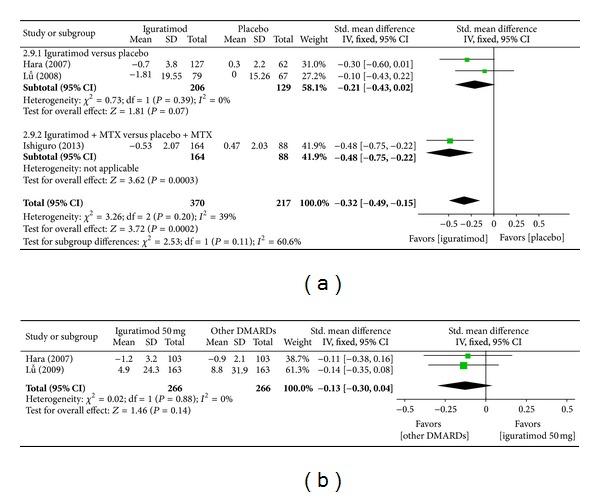
(a) Comparison of CRP score between iguratimod and placebo groups. (b) Comparison of CRP score between iguratimod and other DMARDs (MTX and SASP) groups.

*(h) C-Reactive Protein (CRP)*. Superiority analysis of three studies included [14–16] showed that CRP among patients with RA in the iguratimod group decreased more than that of the placebo group with a significant difference (MD = −0.32, 95% CI: −0.49, −0.15 in Figure 11(a)). Noninferiority analysis of two studies included [14, 15] showed that CRP among patients with RA in the iguratimod group and in the other DMARDs (MTX and SASP) group did not differ significantly (MD = −0.13, 95% CI: −0.30, 0.04 in Figure 11(b)). 

**Figure 12:**
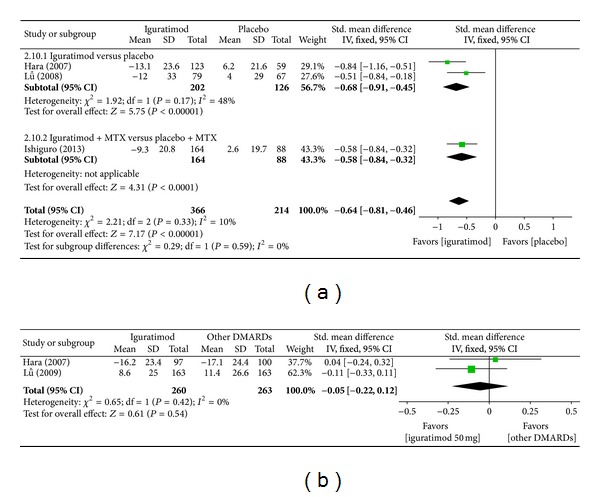
(a) Comparison of ESR score between iguratimod and placebo groups. (b) Comparison of ESR score between iguratimod and other DMARDs (MTX and SASP) groups.

*(i) Erythrocyte Sedimentation Rate (ESR).* Superiority analysis of three studies included [14–16] showed that ESR among patients with RA in the iguratimod group decreased more than that of the placebo group with a significant difference (MD = −0.64, 95% CI: −0.81, −0.46 in Figure 12(a)). Noninferiority analysis of two studies included [14, 15] showed that ESR among patients with RA in the iguratimod group and in the other DMARDs (MTX and SASP) group did not differ significantly (MD = −0.05, 95% CI: −0.22, 0.12 in Figure 12(b)). 

*(j) Radiographic Change of Bone and Joint Damage.* Radiographic change of bone and joint damage was not analyzed for trials of under one year of duration.

**Figure 13:**
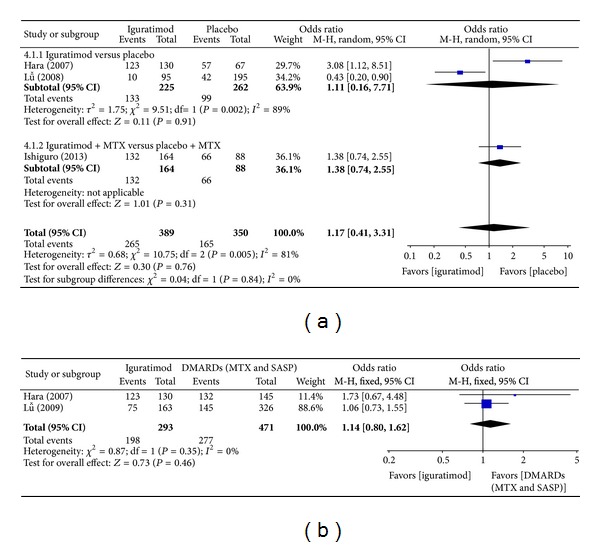
(a) Comparison of adverse events between iguratimod and placebo groups. (b) Comparison of adverse events between iguratimod and other DMARDs (MTX and SASP) groups.

*(2) Secondary Outcomes.* Adverse events of the four studies included [2, 14–16] were described in detail. Pooled analysis of these studies showed no significant difference between the iguratimod group and the placebo group (RR = 1.17, 95% CI: 0.41, 3.31 in Figure 13(b)) or between the iguratimod group and the other DMARDs group (MTX and SASP) (RR = 1.14, 95% CI: 0.80, 1.62 in Figure 13(b)). Adverse events mainly included leucopenia, abnormal level function, upper digestive tract disorder, skin rash, or pruritus. No fatal adverse events were reported.

*(3) Quality of GRADE Evidence. *Assessment for quality of evidence was made using GRADE profiler software recommended by the Cochrane Collaboration. The result of assessment for the four studies included [2, 14–16] was moderate quality (Tables 2 and 3).

### 3.2. Discussion

This systematic review included four eligible studies [2, 14–16]. In three studies, superiority pooled analysis of outcomes showed a significant difference between the iguratimod group and the placebo group with RA. Results showed that iguratimod obviously improved ACR20 and ACR70 at 24 weeks, and patients' health assessment questionnaire (HAQ) score showed reduced rest pain, tender joint count, and swollen joint count; lowered CRP level and ESR; and lowered physician and patient global assessment level of disease activity, but ACR50 at 24 weeks has no significant difference. In two studies [14, 15], noninferiority pooled analysis showed no significant difference between the iguratimod group and the other DMARDs (MTX and SASP) group. Iguratimod was not superior to the other DMARDs (MTX and SASP) in treating RA. All of the four studies [2, 14–16] reported adverse events of iguratimod in treating RA. No significant difference was found in adverse events between the iguratimod group and the placebo group (RR = 1.38, 95% CI: 0.74, 2.55) or the iguratimod group and the other DMARDs (MTX and SASP) group (RR = 1.14, 95% CI: 0.80, 1.62). Major adverse events included hepatic dysfunction, leucopenia, upper digestive tract disorder, skin rash, and pruritus. No fatal adverse events were reported. A long-term clinical study by Hara on iguratimod intake in RA patients for 1 and 2 years showed that iguratimod had the same efficacy and adverse events as those of SASP [19]. Such finding was confirmed by this systematic review.

The units carrying out studies included are all general hospitals or good clinical practice bases of medical universities that have high scientific research diathesis and abilities. Duration and dosage of iguratimod had homogeneity. The evidence of the studies included is moderate quality by carrying out GRADE profiler 3.6 software in spite of some degree-selective biases and no intent-to-treat analyses.

This systematic review has two limitations. First, both the number and the sample size of the studies included are too small. Second, these studies were conducted either in China or in Japan. Similar trials in other countries and regions were not indentified in this review, thus making our review results not representative enough.

In the four studies included [2, 14–16], two described in detail the randomization procedure, allocation concealment, and blinding. We ascertained the validity of the two studies [14, 15] by telephone. For the other two studies [14, 16], we could not reach the authors by email to validate the randomization procedure, allocation concealment, and blinding. Two studies [14, 16] did not have intent-to-treat analysis, and we speculated that participants enrolled were included because of incomplete data. This shortage of two studies [15, 16] made it difficult for us to assess the rationality of study design, and reliability and validity of study results, to some degree, have affected the application of evidence. We hope that future researchers can try to avoid such weaknesses.

## 4. Conclusions

In summary, this systemic review shows that iguratimod is relatively safe and effective in treating RA and its efficacy is the same as that of MTX and SASP. Nevertheless, due to methodological defects, small number, and small sample size of the studies included, we still do not know whether iguratimod is as effective as other DMARDs besides MTX and SASP. We suggest that more high-quality and large-scaled RCTs should be done to determine the efficacy and safety of iguratimod for RA and whether iguratimod is as effective as other DMARDs besides MTX and SASP.

## Figures and Tables

**Figure 2:**
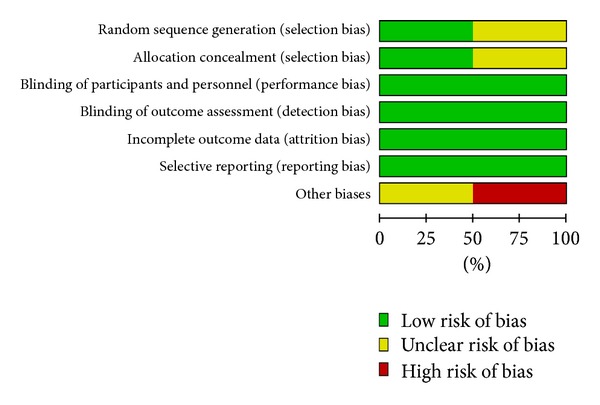
Risk of bias in the 4 studies included.

**Figure 3:**
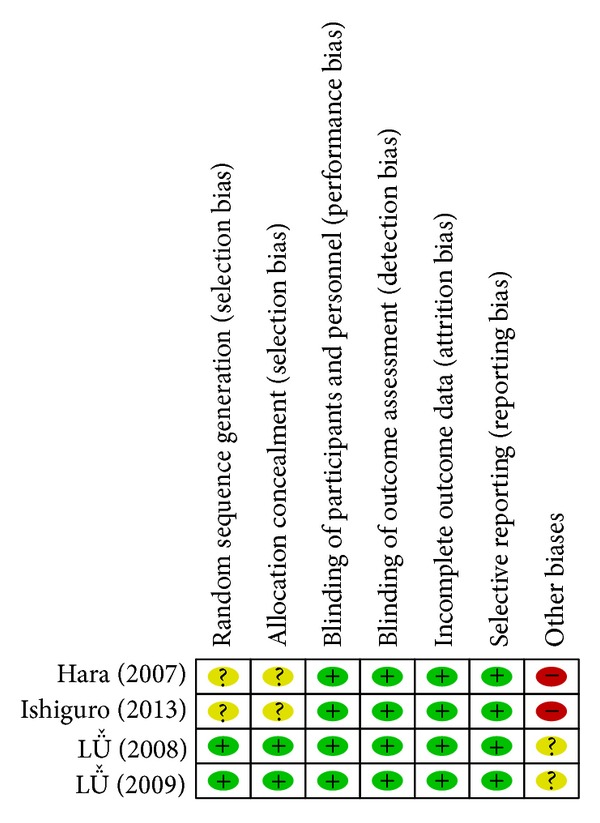
Summary of risk bias in the 4 studies included.

**Table 1:** Characteristics of the included studies with iguratimod (T-614) for rheumatoid arthritis.

Study	Method	Participants	Intervention	Duration	Outcomes	Allocation concealment
Hara et al. (2007) [14]	Double-blind, randomized, placebo-controlled trial	Country: Japan Site: multicenter Number: 376 Iguratimod group (*n* = 147) SASP group (*n* = 156) Placebo group (*n* = 73) Sex: female/male = 306 : 70	T-614 group: iguratimod 25 mg daily for the first 4 weeks and 50 mg daily for the subsequent 24 weeks SASP group: salazosulfapyridine (SASP) 1000 mg daily Placebo group: placebo tablets	28 weeks of clinical assessment at 0, 4, 6, 12, 18, 24, and 28 weeks.	ACR20, ACR50, tender joint count, swollen joint count, rest pain, physician's and patient's global assessment of disease activity (VAS, mm), physician's global assessment of disease activity, HAQ score, ESR, CRP, and adverse events.	Described in article but not validated because the author could not be reached via email.

Lü et al. (2008) [2]	Double-blind, randomized, placebo-controlled trial	Country: China Site: multicenter Number: 280 T-614 group 1 (*n* = 93) T-614 group 2 (*n* = 92) Placebo group (*n* = 95) Sex: female/male = 231 : 49	Group 1: T-614 25 mg daily for the first 4 weeks and 50 mg daily for the subsequent 20 weeks Group 2: T-614 50 mg daily Placebo group: placebo tablets	24 weeks of clinical assessment at 0, 2, 4, 6, 12, 18, and 24 weeks.	ACR20, ACR50, ACR70, tender joint count, swollen joint count, tender joint score (TJS), swollen joint score (SJS), rest pain, duration of morning stiffness, grip strength, physician and patient global assessment of disease activity, ESR, CRP, rheumatoid factor, HAQ score, radiological damage, and adverse events.	Detailed in article and validity ascertained through telephone.

Lü et al. (2009) [15]	Double-blind, randomized, controlled trial	Country: China Site: multicenter Number: 489 T-614 group 1 (*n* = 163) T-614 group 2 (*n* = 163) MTX group (*n* = 163) Sex: female/male = 418 : 81	T-614 group 1: T-614 25 mg daily for the first 4 weeks and 50 mg daily for the subsequent 20 weeks T-614 group 2: T-614 50 mg per day MTX group: MTX 10 mg weekly for the first 4 weeks and 15 mg weekly for the subsequent 20 weeks	24 weeks of clinical assessment at 0, 4, 10, 17, and 24 weeks.	ACR20, ACR50, ACR70, tender joint count, swollen joint count, tender joint score (TJS), swollen joint score (SJS), rest pain, duration of morning stiffness, grip strength, physician and patient global assessment of disease activity, ESR, CRP, and adverse events.	Detailed in article and validity ascertained through telephone.

Ishiguro (2013) [16]	Double-blind, randomized, controlled trial	Country: Japan Site: multicenter Number: 252 T-614 + MTX group (*n* = 164) MTX + placebo group (*n* = 88) Sex: female/male = 204 : 48	T-614 + MTX group: T-614 25 mg daily for the first 4 weeks and 50 mg daily for the subsequent 20 weeks Placebo + MTX group: MTX at low dosages of 6 or 8 mg weekly and folic acid at dosage of 5 mg weekly	28 weeks of clinical assessment at 0, 4, 6, 8, 20, 12, 16, 20, and 24 weeks.	ACR20, ACR50, ACR70, tender joint count, swollen joint count, patient's and physician's global assessment of disease activity, HAQ score, DAS28-CRP, ESR, CRP, and adverse events.	Described in article but not validated because the author could not be reached via email.

**Table 2:** Summary of the findings for the main comparison: iguratimod compared to placebo for rheumatoid arthritis.

Outcomes	Illustrative comparative risks* (95% CI)		Relative effect (95% CI)	No. of participants (studies)	Quality of the evidence (GRADE)	Comments
	Assumed risk	Corresponding risk				
	Placebo	Iguratimod				
ACR20/24 weeks	*219 per 1000 *	*514 per 1000* (398 to 660)	RR 2.35 (1.82 to 3.02)	636 (3 studies)	*⊕⊕* *⊕⊝*moderate^1^	Important
Tender joint count		The mean tender joint count in the intervention groups was *−0.44 lower* (−0.61 to −0.27 lower)		593 (3 studies)	*⊕⊕* *⊕⊝*moderate^1^	Important
Swollen joint count		The mean swollen joint count in the intervention groups was *−0.49 lower* (−0.66 to −0.32 lower)		592 (3 studies)	*⊕⊕* *⊕⊝*moderate^1^	Important
Assessment of rest pain		The mean assessment of rest pain in the intervention groups was *−0.71 lower* (−0.89 to −0.54 lower)		590 (3 studies)	*⊕⊕* *⊕⊝*moderate^1^	Important
Physician global assessment of disease activity		The mean physician global assessment of disease activity in the intervention groups was *−0.74 lower* (−0.93 to −0.55 lower)		592 (3 studies)	*⊕⊕* *⊕⊝*moderate^1^	Important
Patient global assessment of disease activity		The mean patient global assessment of disease activity in the intervention groups was *−0.58 lower* (−0.80 to −0.36 lower)		591 (3 studies)	*⊕⊕* *⊕⊝*moderate^1^	Important
HAQ score		The mean HAQ score in the intervention groups was *−0.67 lower* (−0.84 to −0.50 lower)		591 (3 studies)	*⊕⊕* *⊕⊝*moderate^1^	Important
CRP		The mean CRP in the intervention groups was *−0.31 lower* (−0.53 to −0.09 lower)		587 (3 studies)	*⊕⊕* *⊕⊝*moderate^1^	Important
ESR		The mean ESR in the intervention groups was *−0.64 lower* (−0.82 to −0.45 lower)		530 (3 studies)	*⊕⊕* *⊕⊝*moderate^1^	Important

*The basis for the assumed risk (e.g., the median control group risk across studies) is provided in footnotes. The corresponding risk (and its 95% confidence interval) is based on the assumed risk in the comparison group and the relative effect of the intervention (and its 95% CI). CI: confidence interval; RR: risk ratio; MTX: methotrexate; SASP: salazosulfapyridine.

GRADE working group grades of evidence.

High quality: further research is very unlikely to change our confidence in the estimate of effect.

Moderate quality: further research is likely to have an important impact on our confidence in the estimate of effect and may change the estimate.

Low quality: further research is very likely to have an important impact on our confidence in the estimate of effect and is likely to change the estimate.

Very low quality: we are very uncertain about the estimate.

^1^Two studies had unclear selective biases and no intent-to-treat analyses.

**Table 3:** Summary of the findings for the main comparison: iguratimod compared to the other DMARDs (MTX and SASP) for rheumatoid arthritis.

Outcomes	Illustrative comparative risks* (95% CI)		Relative effect (95% CI)	No. of participants (studies)	Quality of the evidence (GRADE)	Comments
	Assumed risk	Corresponding risk				
	Other DMARDs (MTX and SASP)	Iguratimod				
ACR20/24 weeks	*581 per 1000 *	*505 per 1000* (435 to 592)	RR 0.87 (0.75 to 1.02)	533 (2 studies)	*⊕⊕* *⊕⊝*moderate^1^	Important
Tender joint count		The mean tender joint count in the intervention groups was *−0.01 lower* (−0.18 lower to 0.16 higher)		533 (2 studies)	*⊕⊕* *⊕⊝*moderate^1^	Important
Swollen joint count		The mean swollen joint count in the intervention groups was *−0.15 lower* (−0.32 lower to 0.02 higher)		533 (2 studies)	*⊕⊕* *⊕⊝*moderate^1^	Important
Assessment of rest pain		The mean assessment of rest pain in the intervention groups was *−0.10 lower* (−0.27 lower to 0.07 higher)		530 (2 studies)	*⊕⊕* *⊕⊝*moderate^1^	Important
Physician global assessment of disease activity		The mean physician global assessment of disease activity in the intervention groups was *−0.03 higher* (−0.14 lower to 0.20 higher)		533 (2 studies)	*⊕⊕* *⊕⊝*moderate^1^	Important
Patient global assessment of disease activity		The mean patient global assessment of disease activity in the intervention groups was *−0.05 lower* (−0.22 lower to 0.12 higher)		531 (2 studies)	*⊕⊕* *⊕⊝*moderate^1^	Important
HAQ score		The mean HAQ score in the intervention groups was *−0.09 higher* (−0.08 lower to 0.26 higher)		533 (2 studies)	*⊕⊕* *⊕⊝*moderate^1^	Important
CRP		The mean CRP in the intervention groups was *−0.13 lowe*r (−0.3 to 0.04 lower)		532 (2 studies)	*⊕⊕* *⊕⊝*moderate^1^	Important
ESR		The mean ESR in the intervention groups was *−0.05 lower* (−0.22 lower to 0.12 higher)		523 (2 studies)	*⊕⊕* *⊕⊝*moderate^1^	Important

*The basis for the assumed risk (e.g., the median control group risk across studies) is provided in footnotes. The corresponding risk (and its 95% confidence interval) is based on the assumed risk in the comparison group and the relative effect of the intervention (and its 95% CI). CI: confidence interval; RR: risk ratio; MTX: methotrexate; SASP: salazosulfapyridine.

GRADE working group grades of evidence.

High quality: further research is very unlikely to change our confidence in the estimate of effect.

Moderate quality: further research is likely to have an important impact on our confidence in the estimate of effect and may change the estimate.

Low quality: further research is very likely to have an important impact on our confidence in the estimate of effect and is likely to change the estimate.

Very low quality: we are very uncertain about the estimate.

^1^Random sequence of one study cannot mention how to be generated in detail, and no intent-to-treat analysis was included.
